# The Impact of the COVID-19 Pandemic on Otitis Media

**DOI:** 10.3390/v14112457

**Published:** 2022-11-06

**Authors:** Soo-Young Choi, Dong-Keon Yon, Yong-Sung Choi, Jinseok Lee, Ki-Ho Park, Young-Ju Lee, Sung-Soo Kim, Sang-Hoon Kim, Seung-Geun Yeo

**Affiliations:** 1 Department of Otorhinolaryngology Head & Neck Surgery, Kyung Hee University School of Medicine, Kyung Hee University Medical Center, Seoul 02447, Korea; 2 Center for Digital Health, Medical Science Research Institute, Kyung Hee University School of Medicine, Kyung Hee University Medical Center, Seoul 02447, Korea; 3 Department of Pediatrics, Kyung Hee University School of Medicine, Kyung Hee University Medical Center, Seoul 02447, Korea; 4 Department of Biomedical Engineering, Kyung Hee University, Yongin 17104, Korea; 5 Division of Infectious Diseases, Department of Internal Medicine, Kyung Hee University School of Medicine, Kyung Hee University Medical Center, Seoul 02447, Korea; 6 Department of Obstetrics and Gynecology, Kyung Hee University School of Medicine, Kyung Hee University Medical Center, Seoul 02447, Korea; 7 Department of Biochemistry and Molecular Biology, College of Medicine, Kyung Hee University, Seoul 02447, Korea

**Keywords:** COVID-19, otitis media, prevention, treatment

## Abstract

Otitis media is one of the most common diseases in children, with 80% of children experiencing it by the age of three years. Therefore, the resulting social burden is enormous. In addition, many countries still suffer from complications due to otitis media. Meanwhile, COVID-19 has affected many diseases, with otitis media being one of the most strongly affected. This review aims to find out how COVID-19 has affected otitis media and its significance. A series of measures brought about by COVID-19, including emphasis on personal hygiene and social distancing, had many unexpected positive effects on otitis media. These can be broadly classified into four categories: first, the incidence of otitis media was drastically reduced. Second, antibiotic prescriptions for otitis media decreased. Third, the incidence of complications of otitis media was reduced. Fourth, the number of patients visiting the emergency room due to otitis media decreased. The quarantine measures put in place due to COVID-19 suppressed the onset and exacerbation of otitis media. This has great implications for the treatment and prevention of otitis media.

## 1. Introduction

Otitis media (OM) is a group of complex infectious and inflammatory conditions affecting the middle ear, and various subtypes differ in their presentation, associated complications, and treatment. OM represents a spectrum of diseases, including acute otitis media (AOM), chronic suppurative otitis media (CSOM), and otitis media with effusion (OME) [1,2] (Table 1).

Although OM can affect anyone, it is one of the most common infectious diseases in children, and is associated with significant medical resource use, medical visits, and antibiotic prescriptions. In developed countries, 80% of the children experience at least one episode of AOM by their third birthday. OM can occur at any age but is most common between 6 and 24 months of age. OM is a leading cause of medical visits worldwide, and its complications are an important cause of preventable hearing loss, particularly in developing countries. Consequently, the social burden is large [3,4,5,6,7]. Therefore, several measures have been taken to prevent and treat OM at an early stage.

The COVID-19 pandemic, which has lasted for more than two years, has changed daily lives. This has also changed the disease behavior. On 11 March 2020, the WHO declared COVID-19 a pandemic and introduced several measures to prevent its spread, including personal hygiene measures, such as mask-wearing and hand washing. Social distancing was also strongly recommended, and schools and businesses closed in many countries. These changes in daily life reduced close contact between people, which affected the behavior of people with many diseases. It was expected that the incidence of various infectious diseases would be greatly decreased. In particular, mask-wearing and social distancing were expected to significantly impact upper respiratory tract infections transmitted through breathing.

Since OM is mostly caused by the transmission of upper respiratory tract infections along the ear canal, if the incidence of upper respiratory tract infections is reduced, the incidence of OM will also decrease [2,8]. This is why facilities such as day care centers and kindergartens are risk factors for OM among infants and young children who are prone to OM [9]. However, there are no large-scale studies or guidelines for isolating children to prevent and treat OM. Social distancing due to COVID-19 has provided an unanticipated opportunity to examine the impact of social isolation on OM. Many studies have been conducted to scientifically establish this, and various conclusions have been derived. In this review, major studies and their significance are summarized, with the goal of organizing information to facilitate the prevention and treatment of OM.

## 2. The Epidemiology of OM Changed by COVID-19

The COVID-19 pandemic unexpectedly had many positive effects on OM, given the emphasis on personal hygiene and implementation of social distancing. We reviewed several studies, which are briefly summarized in Table 2, Table 3, Table 4 and Table 5. The observed changes can be broadly classified into the following four categories.

### 2.1. Decreased Incidence Rate of OM

A study that analyzed data obtained from children aged 0–17 in Massachusetts, USA, reported that social distancing (SD) reduced the incidence of many infectious diseases, including AOM. This study compared the incidence of infectious diseases between the pre- and post-SD periods. In the post-SD period, upper respiratory and respiratory infections, such as bronchiolitis, common cold, croup, influenza, pharyngitis, pneumonia, sinusitis, and AOM were drastically reduced. The authors speculated that the reduced prevalence of the diseases and the choice not to seek treatment when sick may have caused these outcomes [10].

In one study, the effect of COVID-19 lockdown on the onset of AOM was analyzed using data collected from six centers in Paris, France. This study, which included data from 871,543 children with pediatric department visits, reported that the incidence of AOM was reduced by more than 70% due to lockdown. In this study, infectious diseases such as the common cold, bronchiolitis, and acute gastroenteritis were also reduced by more than 70%, suggesting that lockdown had a strong effect on blocking infectious diseases in children. However, these results may not accurately reflect the actual incidence of infectious diseases in children, given the potential of caregivers to avoid going to the hospital for fear of contracting COVID-19 [11].

A study conducted by the otolaryngology departments of five tertiary referral centers in Italy investigated the effects of the COVID-19 pandemic on OME. The onset of OME in the pre- and post-pandemic periods was diagnosed and compared using tympanometry, tympanic findings, and pure tone audiometry. OME showed a sharp decline during the pandemic. However, there was no significant difference in the resolution and ventilation tube placement rates due to medical therapy in OME before and after the pandemic.

The reduction in the incidence of OME is explained by the reduction in the transmission of viruses, including respiratory syncytial virus, rhinovirus, adenovirus, bocavirus, influenza virus, parainfluenza virus, enterovirus, and human metapneumovirus, which are known causes of upper respiratory infections and AOM, due to restrictive anti-contagion measures, such as lockdown, continuous use of facial masks, SD, and reduction of social activities. Interestingly, during the pandemic, the incidence of OME decreased to a greater extent in children than in adults. The authors speculated that social isolation was stronger for children than for adults, because schools and kindergartens were completely closed [12].

In a prospective longitudinal study conducted at two hospitals in New York, USA, we investigated the effects of pandemic control measures on AOM and nasopharyngeal colonization in children aged 6–36 months. In this study, nasopharyngeal samples were collected from healthy children who underwent medical checkups at 6, 9, 12, 15, 18, 24, and 36 months and from children with AOM. During the pandemic, the proportion of children visiting AOM drastically decreased. Other respiratory diseases showed similar patterns. During the pandemic, there was a reduced detection of *Haemophilus influenzae* and *Moraxella catarrhalis*, but not *Streptococcus pneumoniae*, in nasopharyngeal samples from healthy children. However, there were no differences in the detection ratios of *Haemophilus influenzae*, *Moraxella catarrhalis*, and *Streptococcus pneumoniae* among children with AOM. As children usually acquire potentially pathogenic respiratory bacteria through close contact and fomite exposure, these findings are thought to be due to social distancing during the pandemic. Notably, the detection rate of oxacillin-resistant *Streptococcus pneumoniae* in nasopharyngeal samples increased during the pandemic, the authors speculated this to be related to bacterial virulence and capacity to colonize the nasopharynx with more limited inoculum from host to host in the absence of viral upper respiratory infection [13].

A German study used nationwide data to analyze the changes in the incidence rates of infectious diseases, injuries, chronic diseases, and mental and behavioral disorders during the COVID-19 pandemic in children aged 0–12 years. The database used in this study contained the medical records of almost all German children. During the COVID-19 period, there was a decrease in the incidence of almost all infectious diseases, as well as diseases of the middle ear and mastoid. This is due to social distancing, which was expected to have a particularly stressful effect on children, potentially leading to the deterioration of mental and physical health. However, a German study found that the incidence of injury, chronic diseases, and mental and behavioral disorders caused by social distancing decreased slightly. What is noteworthy here is that, although it was expected that being unable to visit and play with friends would cause increased social stress for children, this was not found in the studied population. The authors speculated that social distancing stress may offset by increasing family time and bonding. Another possibility is that these mental disorders might become a problem only after a certain amount of time post lockdown. Therefore, the authors argued that appropriate social distancing could be effective in reducing infectious diseases, such as diseases of the middle ear and mastoid, without significant side effects and that social distancing could be an appropriate treatment option for children with severe infectious diseases or weakened immune systems. This report could prove to be an important milestone for future treatment policies aimed at these infectious diseases [14] (Table 2).

**Table 2:** Summary of studies on incidence of OM during the COVID-19 pandemic.

Author [Ref.]	Country	Study Design	Study Population	Outcome Measures	Results
Saskia Hullegie et al. [15]	Netherlands	Retrospective observational cohort study Data were obtained from the Julius General Practitioners’ Network. Its database contains anonymously extracted routine health care data from electronic records from 62 general practices in the Utrecht area	All children aged 0–12 registered 1 March 2019–29 February 2020 (preCOVID-19 pandemic) and/or 1 March 2020–28 February 2021 (COVID-19 pandemic) were included. In the pre-COVID-19 period, electronic health record data of 67,245 children aged 0–12 years were available (time point: 1 September 2019) whereas data of 67,134 children were available during the pandemic (time point: 1 September 2020).	Incidence rates per 1000 child years (IR), incidence rate ratios (IRR) and incidence rate differences (IRD) were compared between the two study periods.	OM episodes including acute mastoiditis declined considerably during the COVID-19 pandemic: IR pre COVID-19 vs. COVID-19 for AOM 73.7 vs. 27.1 [IRR 0.37]; for OME 9.6 vs. 4.1 [IRR 0.43]; and for ear discharge 12.6 vs. 5.8 [IRR 0.46]. The absolute number of AOM episodes in which oral antibiotics were prescribed declined accordingly (IRD pre-COVID-19 vs. COVID-19: −22.4 per 1000 child years), but the proportion of AOM episodes with antibiotic prescription was similar in both the periods (47% vs. 46%, respectively).
Jonathan Hatoun et al. [10]	USA	Retrospective observational cohort study Data were obtained from electronic health record data of a large Massachusetts pediatric primary care network that cares for ∼375,000 children.	Children 0 to 17 years of age for the same calendar period in 2019 and 2020 starting from 1 January. The study defined the pre-social distancing (SD) period as calendar weeks 1 to 9 of each respective year; allowed for a 3-week implementation period as SD was enacted in 2020 and defined the post-SD period as calendar weeks 13 to 18, the most recent data available for analysis. The study did not reveal the total number of persons included.	A difference-in-differences regression analysis was performed using a multivariable Poisson regression model with diagnosis count as a function of calendar year, time period (pre-SD versus post-SD), and the interaction between the two.	The diagnosis rate of AOM was significantly lower in the social distancing period (113.4% vs. 11.5%, respectively). A difference-in-differences regression analysis for 2020 vs. 2019 (95% confidence interval) is −85.1 (−86.8 to −83.5).
Mirko Alde et al. [16]	Italy	Retrospective chart-review study Data were obtained from one pediatric outpatient audiology clinic.	All children aged 6 months to 12 years who attended the outpatient clinic for hearing or vestibular disorders during 2 periods before the lockdown, May–June 2019 (n = 350) and January–February 2020 (n = 366), and the period immediately after the lockdown, May–June 2020 (n = 216) were included. Patients with otomicroscopic evidence of ear disease, craniofacial anomalies, a recent history of medical treatment, etc. were excluded.	The study compared the children’s sex and age characteristics and the distribution of the types of tympanograms in the 3 periods.	The prevalence of OME in this clinic population was 40.6% in May–June 2019, 52.2% in January–February 2020, and 2.3% in May–June 2020. Children with chronic OME had a higher rate of disease resolution in May–June 2020 (93.3%) than those examined in May–June 2019 (20.7%).
Giannicola Iannella et al. [12]	Italy	Retrospective chart review study Data were obtained from five otolaryngology departments of tertiary referral centers.	A total of 1214 patients were included, 526 adults and 688 children between 1 March 2018 and 1 March 2021. In all centers, OME diagnosis were performed according to the commonly recognized OME diagnostic criteria. Patients with otomicroscopic evidence of ear disease, craniofacial anomalies, follow-up loss, etc. were excluded.	To estimate the reduction of OME incidence in children and adults during the COVID-19 pandemic period all patients initially enrolled were divided into three groups according to the following time span. The percent variance of OME incidence between the different time periods was calculated: Group 1—patients with OME diagnosis achieved between 1 March 2018 and 1 March 2019 (not pandemic period). Group 2—patients with OME diagnosis achieved between 1 March 2019 and 1 March 2020 (not pandemic period). Group 3—patients with OME diagnosis achieved between 1 March 2020 and 1 March 2021 (COVID-19 pandemic period).	In the non-pandemic periods (group 1 and 2), the incidence of OME in the five referral centers considered was similar, with 482 and 555 diagnosed cases, respectively. In contrast, the OME incidence in the same centers, during the pandemic period (group 3) was clearly reduced with a lower total number of 177 cases of OME estimated. Percentage variation in OME incidence between the first non-pandemic year considered (group 1) and the pandemic period (group 3) was 63 and 3%, with 305 fewer cases in group 3 compared to group 1. Similarly, comparing the second non-pandemic year (group 2) and the pandemic year (group 3) the percentage variation of OME incidence was 68 and 1%, with 305 fewer cases in group 3 compared to group 2.
Ravinder Kaur et al. [13]	USA	Ongoing prospective longitudinal study Date were obtained from two participating hospital-affiliated pediatric clinical practices	The child population of the two clinics was 12,512 children. All children were 6–36 months old. During the pandemic (from 15 March to 31 December 2020), 258 infection visits occurred among 144 pandemic cohort children compared with 687 visits among 215 prepandemic (from 15 March to 31 December 2019) cohort children.	Physician-diagnosed, medically attended infection visits were assessed in two child cohorts. The study called for the collection of nasopharyngeal (NP) samples at scheduled healthy/well-child visits at age 6, 9, 12, 15, 18, 24, and 36 months and middle ear fluid by tympanocentesis when children experienced AOM.	The pandemic cohort included 144 children, while the pre-pandemic cohort included 215 children. The pandemic cohort experienced 1.8-fold less frequent infectious disease visits during the pandemic. Specifically, visits for AOM were 3.7-fold lower. Compared the isolation rate from the NP in the two cohorts, detection of *Haemophilus influenza* and *Moraxella catarrhalis* significantly decreased during the pandemic (*p* < 0.0001) but not for *Streptococcus pneumoniae*. In contrast, isolation of *Streptococcus pneumoniae*, *Haemophilus influenza* and *Moraxella catarrhalis* from the NP at onset of AOM, when clinical viral upper respiratory infection was concurrently present, did not differ between the cohorts. Oxacillin-resistant *Streptococcus pneumoniae* isolates increased (*p* = 0.009) and b-lactamase-producing *Haemophilus influenza* isolates decreased during the pandemic.
Anna M. Rohe et al. [17]	Germany	Retrospective observational cohort study Data were obtained from 146 ENT practices in Germany.	The study included 162,724 patients in Q2 2019, 158,077 in Q3 2019, 128,342 in Q2 2020, and 149,153 in Q3 2020.	The first outcome was the difference in the number of patients with at least one visit to these practices between the second and third quarters of 2019 and the second and third quarters of 2020. The second outcome was the number of patients with new diagnoses per practice, defined as diagnoses not previously documented in the database for a given patient.	The number of patients per practice was significantly lower in Q2 2020 compared to Q2 2019 (879 versus 1108, *p* < 0.001). There were no significant differences when comparing Q3 2020 to Q3 2019 (1022 versus 1083, *p* = 0.261). Diagnoses of OM were significantly decreased in Q2 2020, the COVID-19 pandemic compared to Q2 2019 (43% decrease). There was still a significant decrease in patient numbers for otitis media (25% decrease) in Q3 2020 compared to Q3 2019.
Natasha Quraishi et al. [18]	UK	Retrospective observational cohort study Data were obtained from three large National Health Service hospitals (1 district general hospital and 2 tertiary centers) covering a catchment population of over 2 million in central England, UK.	A total of 1864 adult admissions in the 2019–2020 period and 791 adult admissions in 2020–2021 period for the ENT infections. Patients aged 16 and older admitted to hospital for ENT infections were included.	Adult hospital admissions for ENT infections over a 12-month period (March 2019 to February 2020 inclusive) before the COVID-19 pandemic were compared with a 12-month period from 23 March 2020 (when the UK government implemented the first national lockdown) to March 2021 inclusive. The main outcome measures were the number of adult hospital admissions.	There was a significant total reduction of 40 admissions (26.85%) for acute otitis media in the 2020–2021 period compared with the 2019–2020 period (*p* = 0.01; RR 1.37, 95% confidence interval [1.07, 1.75]). Two centers, A and C, showed a reduction in admissions for acute otitis media (A: 14 (34.15%); C: 26 (25%)), whereas B showed no change acute otitis media requiring admission.

### 2.2. Decreased Antibiotic Prescription for OM

A study of 405,688 people in the Netherlands found changes in the incidence of infectious diseases during the COVID-19 lockdown. Lockdown was found to reduce various infectious diseases, such as skin and gastrointestinal infections. Notably, the most prominently reduced infectious diseases were respiratory or ear infections. Among the various age groups studied in this work, the respiratory/ear infection frequency decreased most markedly in the 0–12-year-old group and increased slightly in the 41–65-year-old group. The total antibiotic prescription rate for respiratory/ear infections decreased from 21% to 13%. This is interpreted as the result of the lockdown due to COVID-19. This observation contrasted with reports from other countries, which found increases in the prescription rate of antibiotics, but the authors explained that this was due to differences in national health systems, etc. [19] (Table 3).

**Table 3:** Summary of studies on antibiotic prescriptions for OM during the COVID-19 pandemic.

Author [Ref.]	Country	Study Design	Study Population	Outcome Measures	Results
Alma C. van de Pol et al. [19]	Netherlands	Retrospective observational cohort study Date were obtained from pseudonymized routine health care from over 70 primary care practices located in the city of Utrecht and its surrounding areas.	From March through to May 2019 and 2020, 389,708 and 405,688 patients (49% male) were registered in the Julius General Practitioners’ Network practices, respectively. In 2019, 40,219 consultations were extracted, which related to 27,263 infectious disease episodes. In 2020, 37,604 consultations and 23,442 related episodes were found. The overall antibiotic prescription rate was 27% in 2019 and 23% in 2020.	The following outcomes were calculated: (1) the total number of infectious disease episodes recorded from March through May for 2019 and 2020; (2) the number of episodes treated with at least one antibiotic; and (3) the antibiotic prescription rate (proportion of episodes treated with at least one antibiotic). Relative risks were calculated by dividing the risk in 2020 by the risk in 2019. The course of outcomes (1) and (2) over time were determined per week for each infection type. Respiratory/ear infections outcomes (1) and (2) were determined separately per age category of 0–12, 13–40, 41–65, and older than 65 years of age.	Respiratory/ear infection episodes decreased in the youngest and oldest age categories (relative risk, 0.61; confidence interval, 0.58 to 0.64 and relative risk, 0.82; confidence interval, 0.78 to 0.86, respectively), but increased slightly in the 41–65-year-old category (relative risk, 1.14; confidence interval, 1.10 to 1.19). Antibiotic prescriptions for respiratory/ear infections decreased in all age groups, with the largest decrease observed in those aged 0–40 years. Consequently, the antibiotic prescription rate decreased in all age categories.
Sophie E. Katz et al. [20]	USA	Retrospective observational cohort study Data obtained from 4 ambulatory settings affiliated with Vanderbilt University Medical Center: the emergency department, urgent care clinics, primary care clinics, and retail health clinics.	7010 Children (≤18 years) pre-pandemic (P1, 1 March 2019–15 May 2019) and 16,671 children during the early pandemic (P2, 1 March 2020–15 May 2020) were included.	Diagnoses and electronic antibiotic prescriptions were extracted from the electronic medical record. Encounter diagnosis was defined as the International Statistical Classification of Diseases and Related Health Problems, 10th revision diagnosis associated with the antibiotic prescription or the primary encounter diagnosis if no antibiotic was prescribed.	The percent of encounters for infectious diagnoses was lower in P2 (4267/7010, 60.8%) vs. P1 (11,412/16,671, 68.5%) (*p* < 0.001), especially for respiratory diagnoses. The percent of encounters with an antibiotic prescription was lower in P2 than P1 for all encounters (P2: 2240/7010 [32%]; P1 6373/16,671 [38.2%], *p* < 0.001), and among encounters with infectious diagnoses (P2: 1324/2943 [45%]; P1: 3941/7471 [52.8%], *p* < 0.001). In particular, a significant decrease in OM was observed during the early pandemic.

### 2.3. Reduced Emergency Department Visits Due to OM

A study conducted at a tertiary care children’s hospital in Italy analyzed how emergency department (ED) visits changed during the COVID-19 period. The number of children who visited the ED and were hospitalized for various respiratory diseases decreased by 75.8% compared with the total number of children who visited the ED during the same period. Specifically, OM had 162 ED visits per 1000 people before COVID-19, but 26 per 1000 during the COVID-19 period. This indicates that social distancing can strongly block communicable diseases, although the authors cautioned that the differences could be more pronounced because of the reluctance to visit the hospital for fear of COVID-19 infection. According to the results of this study, while the rate of ED visits by critically ill children did not increase during the COVID-19 period, it would be reasonable to interpret that the disease itself decreased [21].

Other studies have reported similar results. A large-scale study based on records of ED visits to 27 children’s hospitals in the United States analyzed how children’s ED visitation behavior changed during the COVID-19 period. The rate of ED visits due to respiratory disease and OM was reduced by 70.0% and 75.1%, respectively [22]. In a similar study of 37 children’s hospitals in the United States, the authors used a predictive model to analyze how much the actual number of children visiting an ED decreased compared with the expected number of ED visits. The results showed a decrease in ED visits due to AOM during the COVID-19 period [23]. A study conducted at two hospitals in Finland also reported a COVID-19-associated decrease in ED visits to children due to AOM [24] (Table 4).

**Table 4:** Summary of studies on visitations to the emergency department for OM during the COVID-19 pandemic.

Author [Ref.]	Country	Study Design	Study Population	Outcome Measures	Results
François Angoulvant et al. [11]	France	A quasi-experimental interrupted time series analysis study Data was based on based on multicenter prospective French surveillance data for pediatric emergency (PED) department visits and related hospital admissions.	A total of 871,543 PED visits in the 6 participating centers from 1 January 2017 to 19 April 2020 were included. Data collected in 2017, 2018, and 2019 were used to generate a model fitting the observed values of the PED visit, allowing us to project the number of PED visits that could have been expected without lockdown.	The main outcome was the evolution of the number of hospital admissions following the French decision to close schools and start a lockdown for the whole country. The secondary outcomes were the number of PED visits for AOM.	A sharp discrepancy was found between expected and observed values after lockdown, reflecting the significant decrease of PED visits (−68.0% [95% confidence interval, −81.2% to −55.8%]) and hospital admission following PED visits (−45.0% [95% confidence interval, −57.0% to −32.4%]) in the lockdown period A significant decrease was found in AOM with a sharp decrease of >70% compared to the expected values.
Gioacchino Andrea Rotulo et al. [21]	Italy	Retrospective chart review study Data obtained from a tertiary care children’s hospital patient visiting the emergency department (ED).	1362 children (age median: 4 years old) were included from 10 March 2020 to 30 April (during the national lockdown) comparing them with 5628 children (age median: 6 years old) of the same time frame of the previous year.	Data were analyzed the overall ED visits comparing 2019 and 2020 cohorts in sex, age, triage code, and outcome. Then, from the total number of accesses to ED, we collected diagnosis of the following acute otitis media.	A total of 1362 children visited the ED during lockdown compared to 5628 during the same period in 2019 (75.8% decrease). The incidence rates and proportions were significantly decreased for otitis (2.6% vs. 16.2%).
Ilari Kuitunen et al. [24]	Finland	Retrospective chart review study Data obtained from 2 Finnish hospitals, and open national registries for communicable diseases.	871 children visited ED 4 weeks before (17 February 2020 to 15 March 2020) and 303 children visited ED 4 weeks after (16 March 2020 to 12 April 2020), the declaration of the Finnish state of emergency on 16 March 2020.	The infections were classified as upper or lower by the International Classification of Diseases, 10th revision, diagnosis of the visit. The infectious disease register is a nationwide register maintained by the Finnish Institute of Health and Welfare.	Before the pandemic, there were 92 children who visited ED with AOM and 32 after the pandemic. The age distribution of patients at both hospitals remained similar before and after the lockdown. There was an overall decrease in the number of hospitalized patients, especially due to respiratory infections after the lockdown. No COVID-19 cases were detected in children in either of the participating hospitals.
Amy M. DeLaroche et al. [25]	USA	Retrospective cross-sectional study Data were obtained from the Pediatric Health Information System, an administrative database including 50 tertiary care children’s hospitals in the United States.	The study included all ED visits during the COVID-19 pandemic (15 March 2020 to 31 August 2020, N = 495,052) and a 3-year comparator period (15 March–31 August 2017–2019, N = 2,733,078) for 27 hospitals with complete administrative and billing data for the study periods.	To account for yearly variation in ED volume and case mix, the study averaged visit numbers for the 3-year comparator period across the same calendar dates as the pandemic period. The study examined primary discharge diagnoses, ED management, disposition, and select quality metrics for each visit.	Sharp declines in ED visits were observed for less urgent conditions, such as OM and upper respiratory infection (75.1% and 69.6% decreases, respectively). The percentage of visits to ED for total diseases of the ear and mastoid process was reduced by 68.0%. Among them, OM decreased by 75.1%, other specified and unspecified disorders of the ear by 42.5%, and diseases of middle ear and mastoid (except otitis media) by 65.3%.
Sriram Ramgopal et al. [23]	USA	Retrospective cross-sectional study Data were obtained from 37 freestanding and non-freestanding pediatric hospitals which contribute data to the Pediatric Health Information System Constructed ensemble forecasting models using data from 2010–2019 and compared the forecasts to the 2020 data.	The study abstracted data from all encounters between 1 January 2010 and 31 December 2020 and with an associated ED charge. (27,874,730 children, median age: 4.8 in 2010–2019, 1,913,085, median age: 5.7 children in 2020, respectively) No exclusions were applied.	The study compared demographics (age, race, ethnicity, payor status, time of year, census region), diagnoses, and measures of acuity (hospitalization, ICU admission, and in-hospital mortality), and abstract based charges between 2010 and 2019 and 2020. The study transformed groupings of ED encounters (by month and year) into a time series object and identified seasonal trends between 2010 and 2019 for all encounters with estimated scatterplot smoothing and results displayed graphically.	Pediatric ED utilization remained low following the COVID-19 pandemic and was below the forecasted utilization for AOM. A total of 816,414 (2.9%) ED encounters for AOM occurred between 2010 and 2019 and a total of 24,322 (1.3%) ED encounters for AOM occurred in 2020.
Sarina Bucher et al. [26]	Switzerland	Retrospective chart review study Data obtained from one tertiary referral center on emergency otorhinolaryngologic consultations.	A total of 495 emergency consultations were recorded during the lockdown (between 16 March 2020 and 26 April 2020); in comparison, there were 886 emergency consultations during the same period in 2019.	Primary outcomes were defined as differences in the number of emergency consultations in 2020 versus 2019.	During lockdown, the largest decreases of consultation numbers were seen for OM and eustachian tube dysfunction. Seventeen patients sought emergency assistance for otitis media in 2020, compared with 83 in 2019 (*p* < 0.001, OR 2.906, 95% confidence interval 1.704–4.957). Only 3 patients with Eustachian tube dysfunction were seen in 2020 versus 23 in 2019 (*p* = 0.007, OR 4.371, 95% confidence interval 1.306–14.631).

### 2.4. Reduced Complications of OM

In an Italian study, the effect of social restrictions on OME was examined. OME was evaluated by tympanometry in 932 children aged 6–12 years who visited a pediatric outpatient audiology clinic in Milan, Italy. The presence or absence of OME was diagnosed based on the tympanic membrane and type B tympanogram findings. The authors compared the onset of OME for consistent periods before and after social restriction and reported that social restriction sharply lowered the incidence of OME. The researchers observed that social restriction had the effect of blocking all infectious diseases and that OM decreased accordingly. In addition, the authors speculated that children with severe or incurable OME had more resolution after their social restriction, which could help inform the treatment of patients with OME in the future [16].

A study conducted in the Netherlands compared the incidence rates of OM and related complications between the COVID-19 pre-pandemic and pandemic periods among children aged 0–12 using a database in the Utrecht area. The study included 67,245 and 67,134 children during the COVID-19 pre-pandemic and pandemic periods, respectively. AOM, OME, and ear discharge decreased dramatically during the pandemic, and the incidence of acute mastoiditis and antibiotic prescription decreased accordingly. The authors mainly attributed the decrease in URI to lockdown and increased hand washing [15].

In a study conducted in Italy, 102 otitis-prone children (with a history of recurrent acute OM defined as ≥3 distinct episodes in 6 months or ≥4 in 12 months) were analyzed for changes in their OM status during the COVID-19 lockdown. The study found that during the lockdown period, 82% of children showed improvement in OM. Spontaneous tympanic membrane perforation episodes, otorrhea episodes, and systemic antibiotic treatment were significantly reduced. The authors speculated that this may have reflected the complete social isolation of the children rather than the avoidance of contact by otitis-prone children. The authors also proposed that lockdown-related improvements in air pollution may have played a role [27] (Table 5).

**Table 5:** Summary of studies on complications of OM during the COVID-19 pandemic.

Author [Ref.]	Country	Study Design	Study Population	Outcome Measures	Results
Sara Torretta et al. [27]	Italy	Retrospective chart review study Date were obtained from tertiary outpatient clinic	A total of 102 children were included. (50% males, mean age was 41.4 ± 14.0 months)	Families of otitis-prone children (i.e., with a history of recurrent acute otitis media defined as ≥3 distinct episodes in 6 months, or ≥4 in 12 months) scheduled for periodic evaluation were contacted through telephone calls by resident physicians. Parents were asked to provide their subjective opinion on their children’s clinical conditions (improved, stable, worsened) during lockdown. The mean number of episodes of acute otitis media without spontaneous tympanic membrane perforation and otorrhea episodes, as well as the number of systemic antibiotic treatments administered was recorded and compared with non-pandemic period.	Most parents (82.3%) declared that their children’s condition had improved during the lockdown, and 16.7% stated that their children’s condition was stable. There was a statistically significant reduction in the mean number of episodes of AOM without spontaneous tympanic membrane perforation, otorrhea, or systemic antibiotic treatment during pandemic period. (0.37 vs. 0.07, 0.48 vs. 0.01, 0.85 vs. 0.09, respectively).
Mara Barschkett et al. [14]	Germany	Retrospective observational cohort study Date obtained from nationwide data of the Kassenärztliche Bundesvereinigung (National Association of Statutory Health Insurance Physicians) in anonymized form for the population of all children with statutory health insurance in Germany with at least one visit to a medical doctor’s office between January 2019 and June 2020.	A total of 8.29 million children (0–15 years) in 2019 and 8.5 million children in 2020 were included. In 2019 and 2020, about 90% of all children between 0 and 15 years were insured via one of the statutory health insurance funds. Thus, the data cover most children living in Germany.	The analyzed dataset includes the number of treatment cases and, at the patient level, the diagnoses documented with ICD-10 codes. The frequencies of outpatient treatment cases and selected diagnoses were evaluated for the second quarter (Q2) of 2019 (control period) for children born in the period of 2007–2018 and for Q2 of 2020 (pandemic period) for children born in the period of 2008–2019.	Among outpatient visits for infectious disorders, there was a particularly marked reduction in OM with other middle ear and mastoid diseases in children aged 1–2 and 3–5 years (22% and 28% decreases, respectively).

### 2.5. Undetermined Whether OM Symptoms Worsened

OM can be caused by the transfer of pathogens from the nasopharynx to the middle ear. Viruses are one of the main causes of OM; they can be the sole cause of OM, or they can promote bacterial infection leading to OM. Viral infection adversely affects the middle ear in various ways, including immune disruption and reduced mucociliary function. The symptoms of OM have been reported to be more severe in patients infected with a virus [28,29]. Similarly, there are reports that OM symptoms are worse if OM develops in individuals with COVID-19. However, it is not yet clear whether SARS-CoV-2 (the virus that causes COVID-19) directly infects the middle ear and causes OM [30,31]. In another study, the symptoms of OM did not differ significantly between patients with and without COVID-19. In addition, another study observed no treatment failure, recurrence, or complications when OM occurred in COVID-19 patients [32]. Therefore, it is not yet known whether the symptoms of OM caused by SARS-CoV-2 infection are more severe or the prognosis is worse than those of OM caused by other viral infections.

## 3. Conclusions

The measures, including emphasis on personal hygiene and social distancing, put in place as a response to COVID-19, had many positive effects on OM. Due to the reduction of upper respiratory tract infections, the incidence of OM decreased drastically, as did the prescription of antibiotics. These changes resulted in a decrease in OM complications and the number of patients visiting the ED for OM.

Interestingly, strict personal hygiene management and social distancing lowered the severity of existing severe OM and complications. This finding suggests that quarantine measures alone, rather than conventional aggressive treatments, can prevent disease progression. Although more studies are needed in the future, the existing findings provide a useful reference for future OM treatment guidelines.

Conversely, some aspects of the measures implemented in response to COVID-19 may have negative effects on OM in the future. Personal protective measures may induce a kind of “immunity debt” in children. The acquired immunity, which must be naturally formed during social activities, is insufficient, which may lead to more frequent and serious development of upper respiratory tract infections and OM in the future [33]. In addition, due to social distancing, essential vaccinations have been delayed. This may increase the risk of outbreaks of various diseases that are preventable by vaccination. OM is one of the diseases affected by these delays, and it is necessary to quickly catch up with the essential vaccination schedule to supplement immunity [34,35].

It is difficult to definitively conclude how COVID-19 has affected OM, especially given that the world is not yet completely free from COVID-19. Based on these meaningful results, further studies are needed in the future.

## Figures and Tables

**Table 1:** Definition of otitis media.

Term	Definition
Otitis media	All types of inflammation of the middle ear.
Acute otitis media	A short-term inflammation of the middle ear, characterized by the rapid onset of signs or symptoms (bulging of the tympanic membrane, ear pain, erythema of the tympanic membrane, acute ear discharge from the tympanic membrane, etc.).
Chronic suppurative otitis media	A persistent inflammatory condition of the middle ear and mastoid associated with a perforated tympanic membrane and persistent long-term ear discharge from the middle ear (no consensus on the duration of ear discharge needed for diagnosis, with recommendations ranging from 2 weeks to at least 3 months).
Otitis media with effusion	The presence of fluid in the middle ear without signs or symptoms of acute ear infection.
Middle ear effusion	Fluid in the middle ear from any cause. Middle ear effusion is present with both OME and AOM and may persist for weeks or months after the signs and symptoms of AOM resolve.

AOM, acute otitis media; OME, otitis media with effusion.

## Data Availability

Not applicable.
